# Supplementary material for: Local auxin biosynthesis acts downstream of brassinosteroids to trigger root foraging for nitrogen
Source: Nat Commun. 2021 Sep 14;12:5437. doi: 10.1038/s41467-021-25250-x (PMC8440578; doi:10.1038/s41467-021-25250-x)
Supplement: Supplementary file 1 — Supplementary Information [file 41467_2021_25250_MOESM1_ESM.pdf]

Supplementary Information

Local auxin biosynthesis acts downstream of brassinosteroids to trigger root foraging for nitrogen

Jia et al.

* Correspondence: Nicolaus von Wirén

E-mail: vonwiren@ipk-gatersleben.de

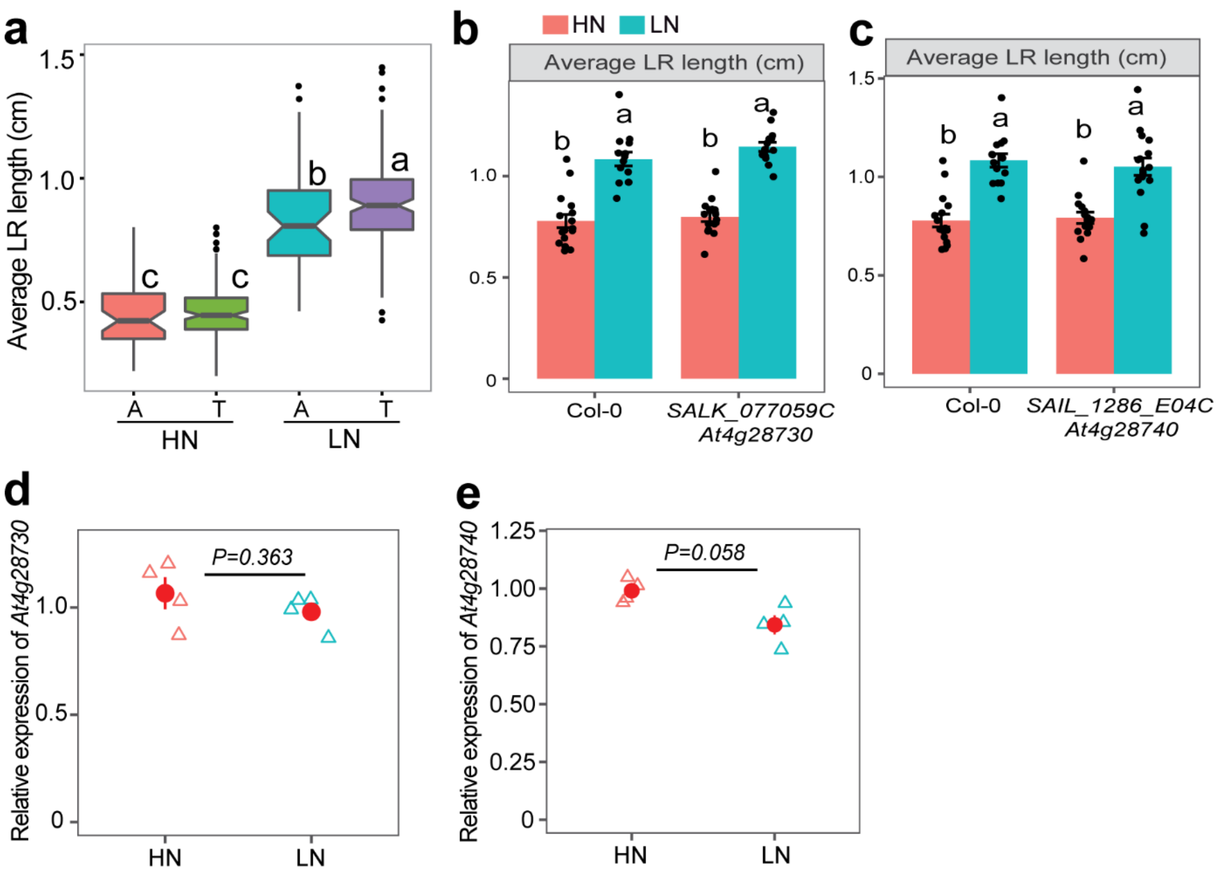

Supplementary Figure 1. Lateral root length of A or T accessions and of T-DNA insertion lines of two genes surrounding the*YUC8* locus. a, Average LR length of accessions with alternative allelic variants for the lead SNP (n = 50 and 150 accessions for A- and T-allele, respectively). Horizontal lines show medians; box limits indicate the 25th and 75th percentiles; whiskers extend to 1.5 times the interquartile range from the 25th and 75th percentiles. **b-c** Average LR length of T-DNA insertion lines for At4g28730 **(b)** and At4g28740 **(c)**. Data of wildtype Col-0 in **b** and **c** are the same because two T-DNA insertion lines were phenotyped together with wildtype Col-0 in the same experiment. Bars represent means ± SEM. Number of individual roots analyzed in HN/LN: n = 16/14 (Col-0), 16/14 (*SALK_077059C*), 15/16 (*SAIL_1286E04C*). Seven-day-old seedlings pre-cultured on 11.4 mM N were transferred to agar media containing either high N (HN, 11.4 mM N) or low N (LN, 0.55 mM N). Average length of LRs was determined after 9 days. Different letters in **a**-**c** indicate significant differences at P< 0.05 according to one-way ANOVA and post-hoc Tukey test. **d-e** Transcript level of *At4g28730* **(d)** and *At4g28740* **(e)** in the roots of Col-0 in response to LN. Root samples for *q*PCR analysis were taken 9 days after transfer to N treatments. Expression levels were assessed in whole roots by *q*PCR analysis and normalized to *ACT2* and *UBQ10*. Each triangle represents the value of one individual biological replicate (n=4). Red dots with crossbars represent means ± SEM. *P* values relate to differences between two N conditions according to Welch's *t*-test.

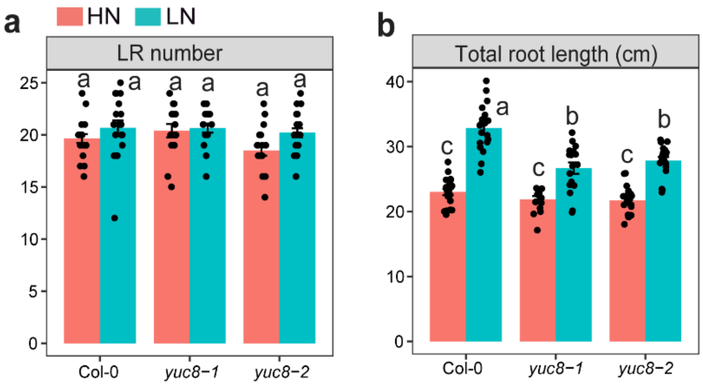

Supplementary Figure 2. Lateral root number and total root length of two *yuc8* mutant lines at high or low N. **a-b** LR number **(a)** and total root length **(b)** of wild type (Col-0) and *yuc8* mutants. Bars represent means ± SEM. Number of individual roots analyzed in HN/LN: *n* = 20/19 (Col-0), 15/17 (*yuc8-1*)*,* 20/20 (*yuc8-2*). Seven-day-old seedlings pre-cultured on 11.4 mM N were transferred to solid agar media containing either high N (HN, 11.4 mM N) or low N (LN, 0.55 mM N). Root system architecture was assessed after 9 days. Different letters indicate significant differences at $P < 0.05$ according to one-way ANOVA and post-hoc Tukey test.

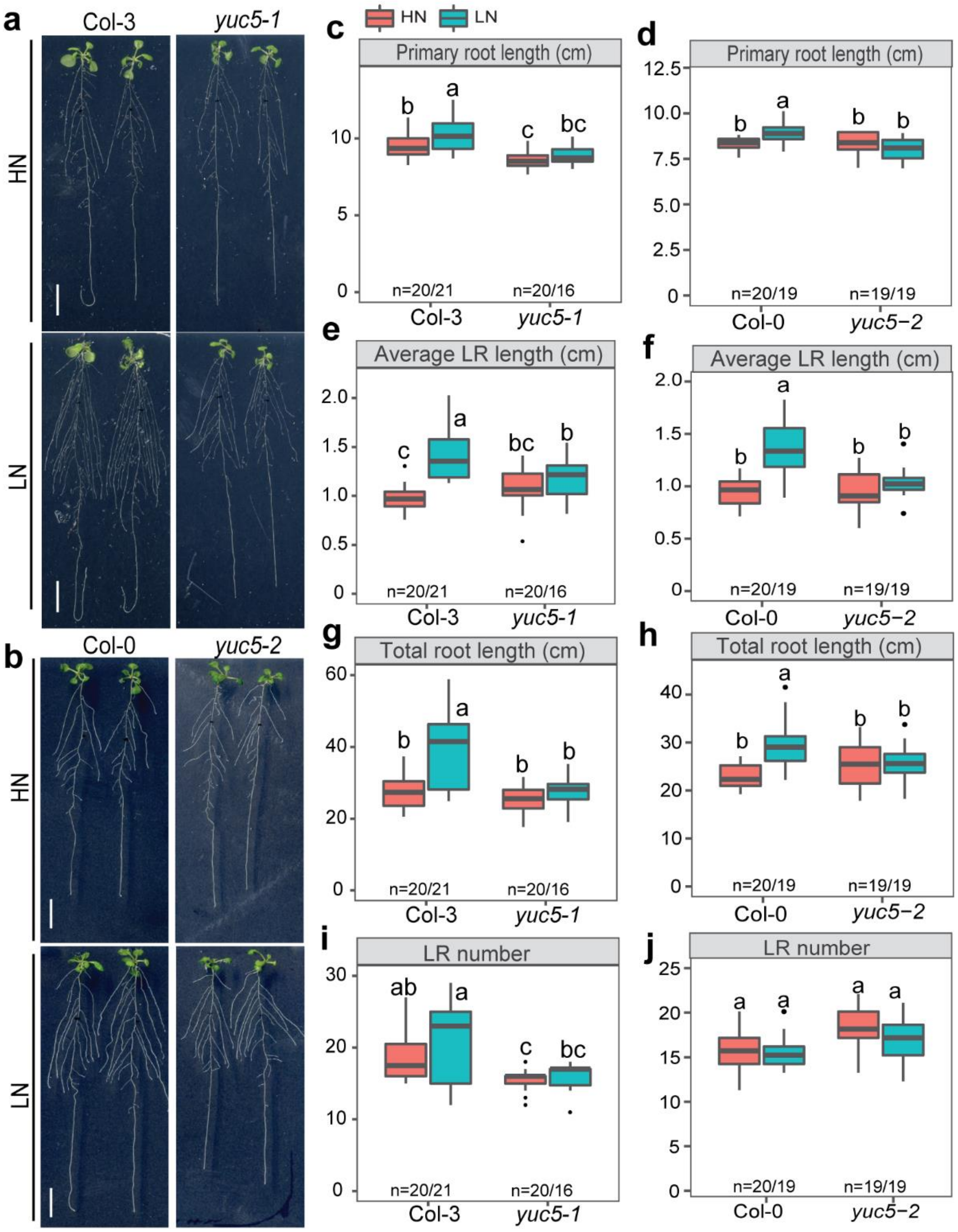

Supplementary Figure 3. Root architectural traits of *yuc5* mutants at high or low N. a-j Appearance of plants **(a,b)**, PR length **(c,d)**, average LR length **(e,f)**, total root length **(g,h)** and LR number **(i,j)** of wild-type (Col-0) and two independent *yuc5* mutants. Seven-day-old seedlings were pre-cultured on 11.4 mM N and then transferred to agar media containing either high N (HN, 11.4 mM N) or low N (LN, 0.55 mM N). Root system architecture was assessed after 9 days. Numbers below each box indicate the number of plants assessed for each genotype under HN and LN. Horizontal lines show medians; box limits indicate the 25th and 75th percentiles; whiskers extend to 1.5 times the interquartile range from the 25th and 75th percentiles. Different letters indicate significant differences at P< 0.05 according to one-way ANOVA and post-hoc Tukey test. Scale bars, 1 cm.

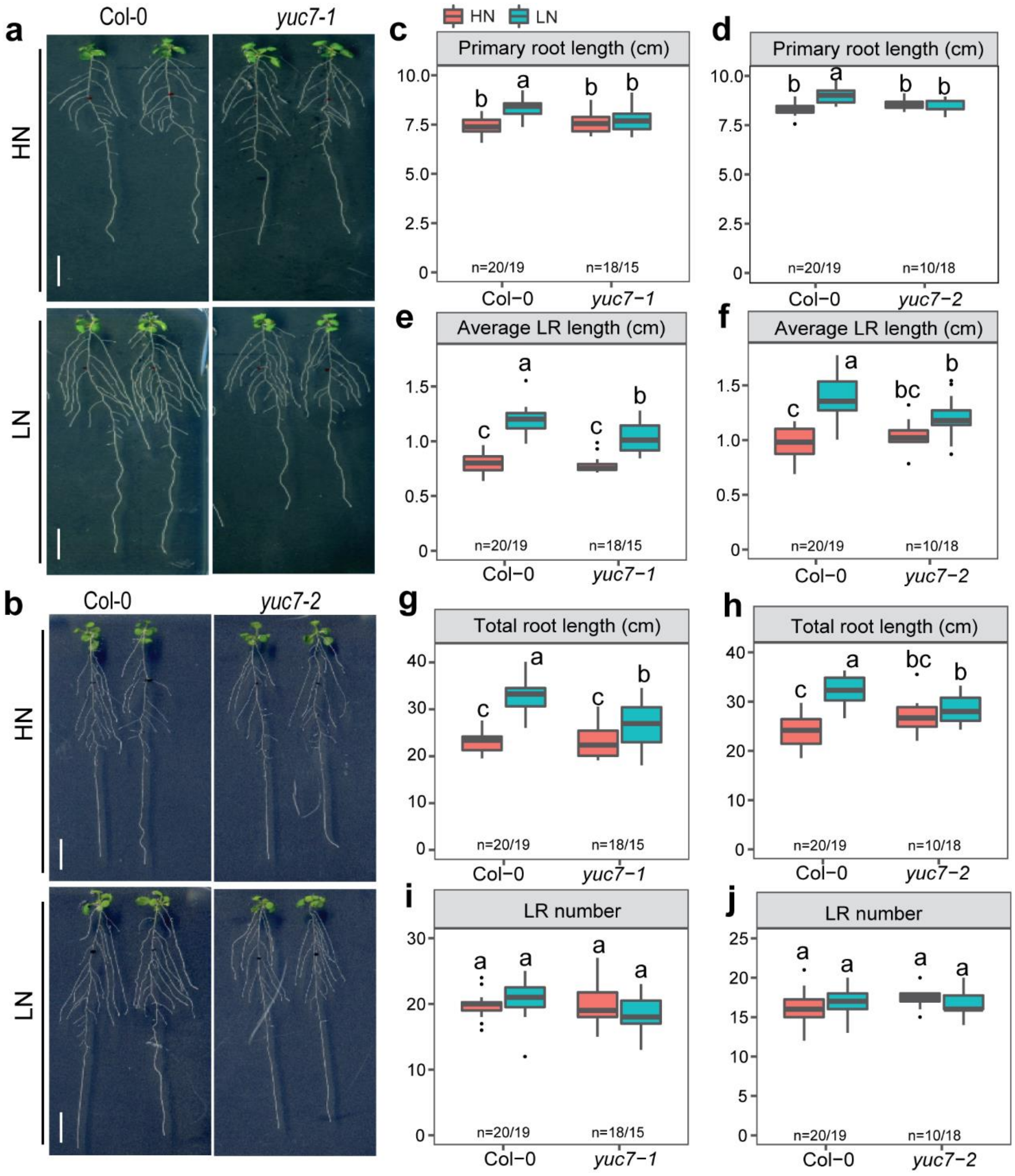

Supplementary Figure 4. Root architectural traits of *yuc7* mutants at high or low N. a-j Appearance of plants **(a,b)**, PR length **(c,d)**, average LR length **(e,f)**, total root length **(g,h)** and LR number **(i,j)** of wild-type (Col-0) and two independent *yuc7* mutants. Seven-day-old seedlings were pre-cultured on 11.4 mM N and then transferred to agar media containing either high N (HN, 11.4 mM N) or low N (LN, 0.55 mM N). Root system architecture was assessed after 9 days. Numbers below each box indicate the number of plants assessed for each genotype under HN and LN. Horizontal lines show medians; box limits indicate the 25th and 75th percentiles; whiskers extend to 1.5 times the interquartile range from the 25th and 75th percentiles. Different letters indicate significant differences at $P < 0.05$ according to one-way ANOVA and post-hoc Tukey test. Scale bars, 1 cm.

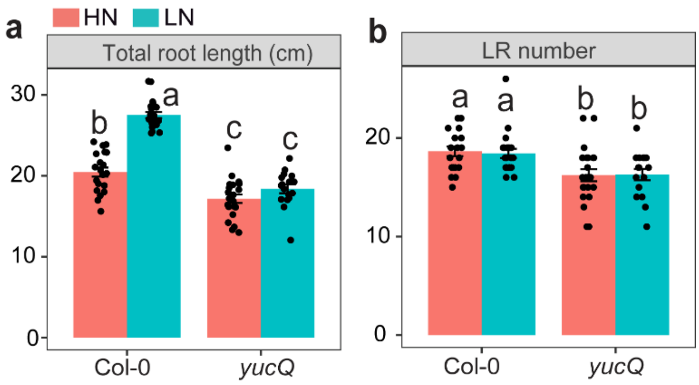

Supplementary Figure 5. Total root length and LR number of the *yucQ* mutant at high or low N. **a-b** Total root length **(a)** and LR number **(b)** of wild type (Col-0) and *yucQ* mutant plants grown under HN or LN. Bars represent means ± SEM. Number of individual roots analyzed in HN/LN: *n* = 20/21 (Col-0) and 22/17 (*yucQ*). Seven-day-old seedlings pre-cultured on 11.4 mM N were transferred to solid agar media containing either high N (HN, 11.4 mM N) or low N (LN, 0.55 mM N). Root architectural traits were assessed after 9 days. Different letters indicate significant differences at $P < 0.05$ according to one-way ANOVA and post-hoc Tukey test.

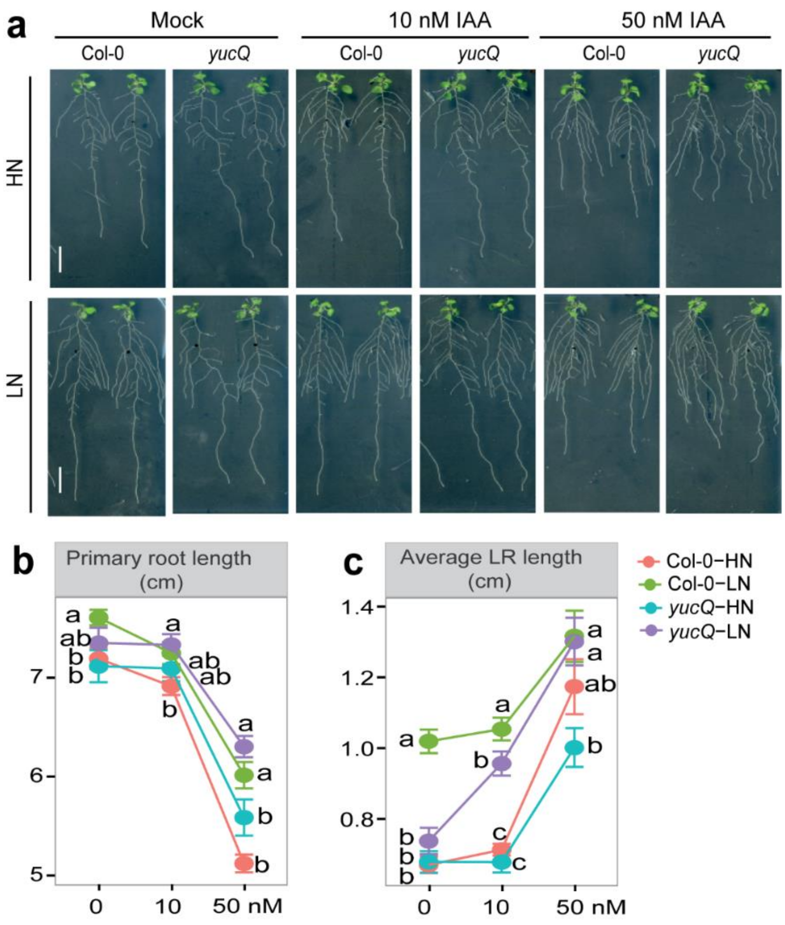

Supplementary Figure 6. Exogenous supply of auxin restores the LR elongation of *yucQ* mutants at low N. a-c Appearance of plants **(a)**, PR length **(b)**, and average LR length **(c)** of wild-type (Col-0) and *yucQ* mutant plants grown at high N (HN, 11.4 mM N) or low N (LN, 0.55 mM N) in the presence or absence of exogenously supplied IAA at indicated concentrations. Lengths of PR and LRs were assessed after 9 days. Dots represent means ± SEM. Number of individual roots analyzed in HN/LN: *n* = 13/15 (mock), 14/13 (10 nM IAA), 15/14 (50 nM IAA) for Col-0; *n* = 15/10 (mock), 12/10 (10 nM IAA), 11/14 (50 nM IAA) for *yucQ*). Different letters indicate significant differences at $P < 0.05$ according to one-way ANOVA and post-hoc Tukey test within each IAA treatment. Scale bars, 1 cm.

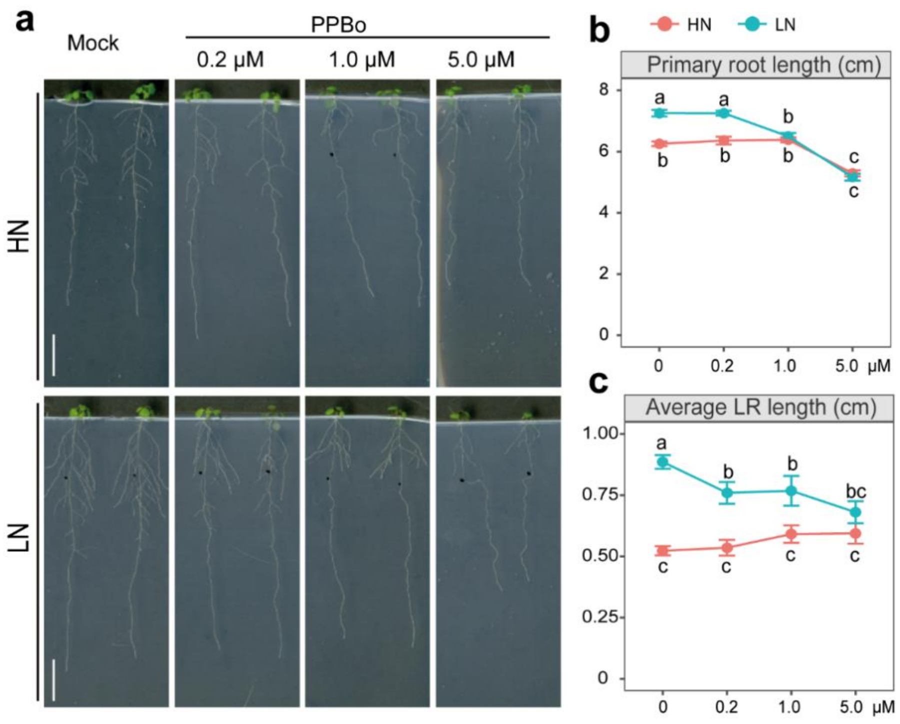

Supplementary Figure 7. Exogenous application of the YUC inhibitor PPBo to roots blocks root elongation at low N. a-c Appearance of plants **(a)**, PR length **(b)**, and average LR length **(c)** of wild-type (Col-0) plants grown at high N (HN, 11.4 mM N) or low N (LN, 0.55 mM N) in the presence or absence of 4-phenoxyphenyl boronic acid (PPBo) at indicated concentrations. Lengths of PR and LRs were assessed after 9 days. Dots represent means ± SEM. Number of individual roots analyzed in HN/LN: *n* = 18/17 (mock), 20/20 (0.2 µM PPBo), 18/17 (1 µM PPBo) and 16/14 (5 µM PPBo). Different letters indicate significant differences at $P < 0.05$ according to one-way ANOVA and post-hoc Tukey test. Scale bars, 1 cm.

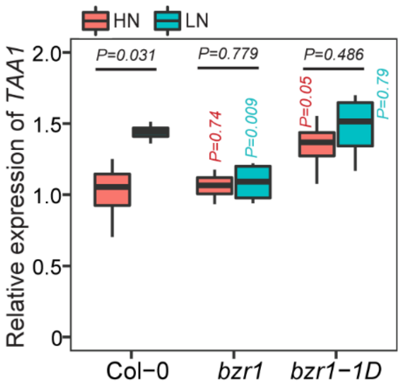

Supplementary Figure 8. *TAA1* is up-regulated by low N in a brassinosteroid signaling-dependent manner. Transcript level of *TAA1* in wild-type (Col-0), *bzr1* and *bzr1-1D* roots at high N (HN, 11.4 mM N) or low N (0.55 mM N) availabilities. Seven-day-old seedlings were pre-cultured on 11.4 mM N and then transferred to agar containing either HN or LN. Samples for *q*PCR analysis were taken 9 days after transfer. Expression levels were normalized to *ACT2* and *UBQ10*. n=4 independent biological replicates. Horizontal lines show medians; box limits indicate the 25th and 75th percentiles; whiskers extend to 1.5 times the interquartile range from the 25th and 75th percentiles. *P* values relate to differences between two N conditions for each genotype (in black) or between wild-type and mutant plants under HN (in red) or LN (in cyan) according to Welch's *t*-test.

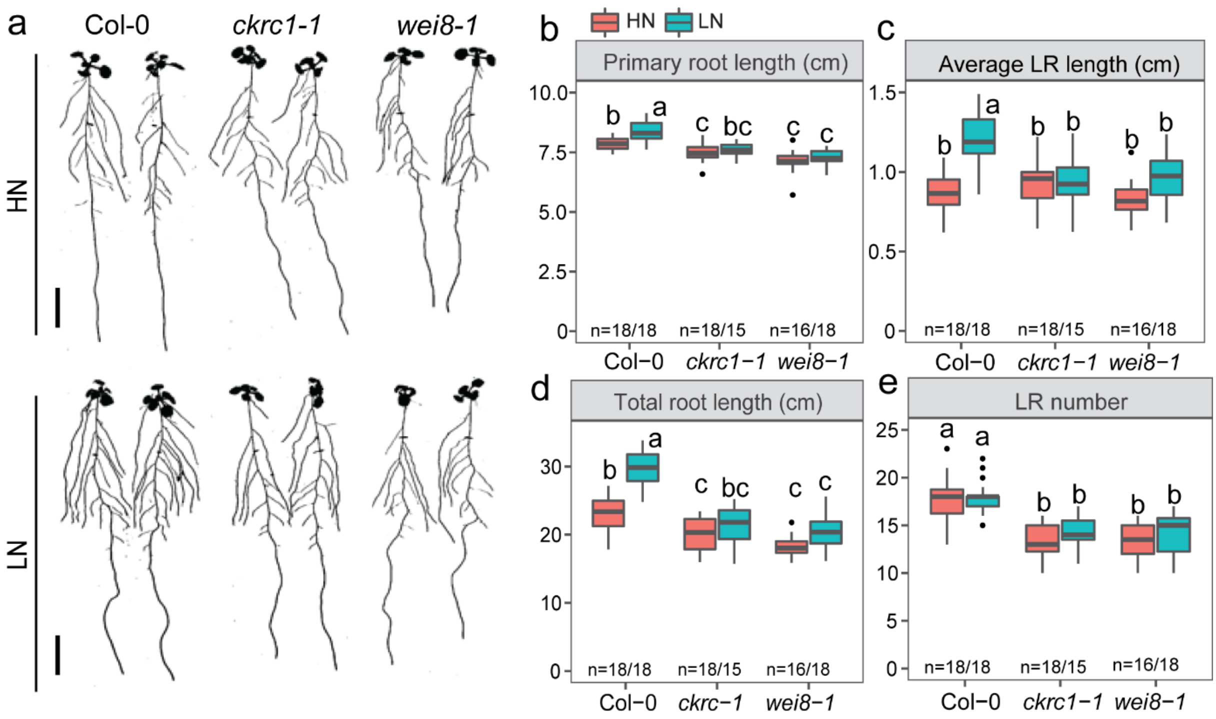

Supplementary Figure 9. Root architectural traits of *TAA1* mutants at high or low N. a-e Appearance of plants **(a)**, PR length **(b)**, average LR length **(c)**, total root length **(d)** and LR number **(e)** of wild-type (Col-0) and the *TAA1* mutants *ckrc1-1* and *wei8-1*. Seven-day-old seedlings were pre-cultured on 11.4 mM N and then transferred to agar media containing either high N (HN, 11.4 mM N) or low N (0.55 mM N). Root system architecture was assessed after 9 days. Numbers below each box indicate the number of plants assessed for each genotype under HN and LN. Horizontal lines show medians; box limits indicate the 25th and 75th percentiles; whiskers extend to 1.5 times the interquartile range from the 25th and 75th percentiles. Different letters indicate significant differences at $P < 0.05$ according to one-way ANOVA and post-hoc Tukey test. Scale bars, 1 cm.

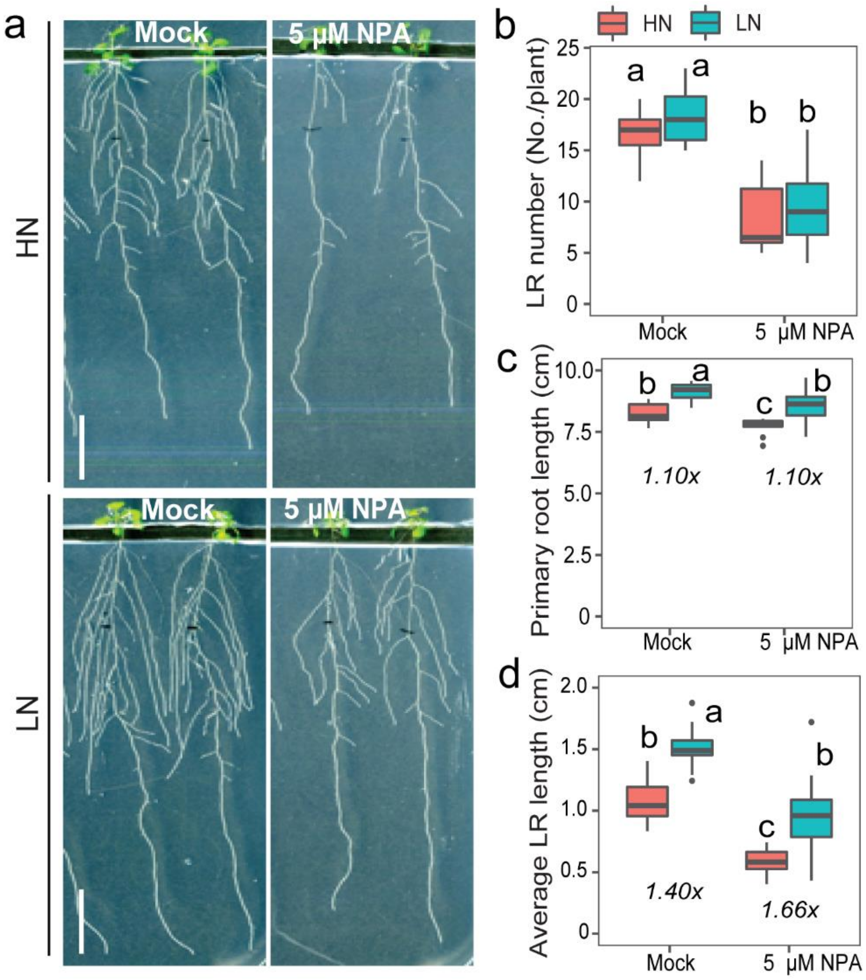

Supplementary Figure 10. Shoot-derived auxin is not critical for the root responses to low N. a-d Appearance of plants **(a)**, LR number **(b)**, primary root length (**c**), and average LR length **(d)** of wild-type (Col-0) plants grown under high N (HN, 11.4 mM N) or low N (LN, 0.55 mM N). The agar segment in the shoot compartment was supplied with 5 µM polar auxin transport inhibitor N-naphthylphtalamic acid (NPA) or the solvent (mock). The number, length of primary root and LRs were assessed after 9 days (*n* = 12 plants). Values in **c** and **d** indicate fold-change increase of primary root and average LR length of LN versus HN. Note that NPA supply to shoots caused the expected decrease in emerged LRs irrespective of the N condition but did not inhibit the low N-induced PR and LR elongation. Horizontal lines show medians; box limits indicate the 25th and 75th percentiles; whiskers extend to 1.5 times the interquartile range from the 25th and 75th percentiles. Different letters indicate significant differences at $P < 0.05$ according to one-way ANOVA and post-hoc Tukey test. Scale bars, 1 cm.

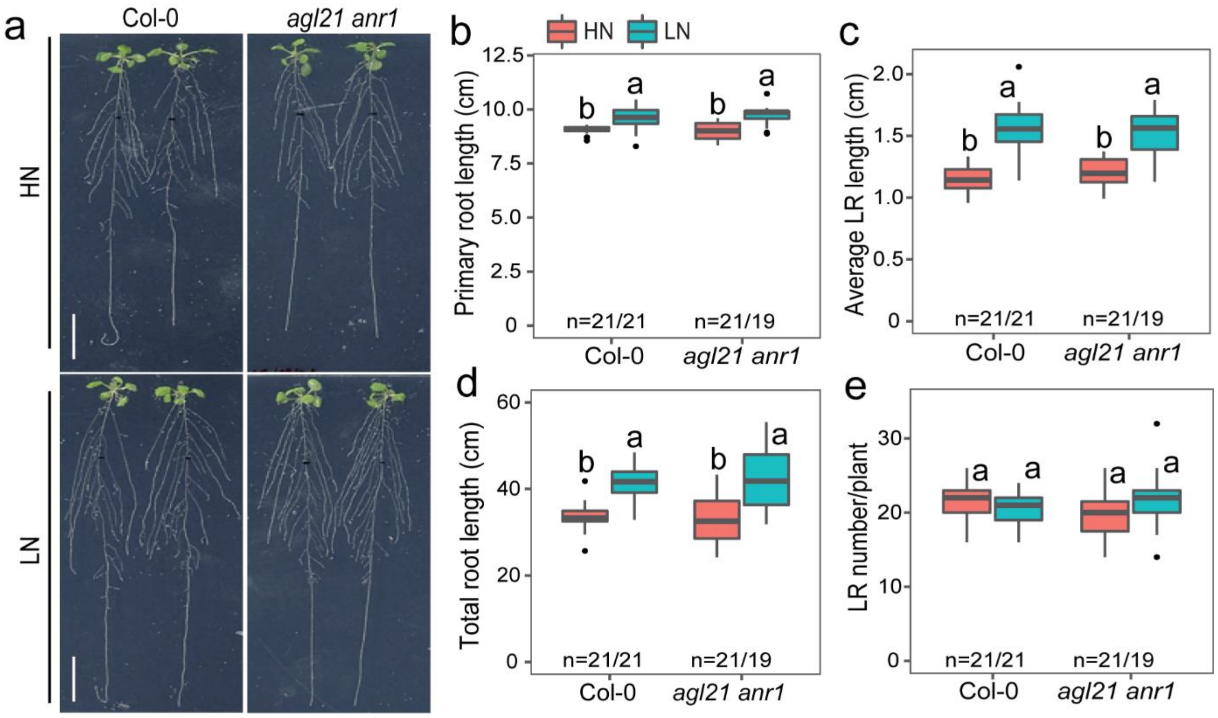

Supplementary Figure 11. Root architectural traits of *agl21 anr1* double mutant at high or low N. a-e Appearance of plants **(a)**, PR length **(b)**, average LR length **(c)**, total root length **(d)** and LR number **(e)** of wild-type (Col-0) and *agl21 anr1* mutant plants. Seven-day-old seedlings were pre-cultured on 11.4 mM N and then transferred to agar media containing either high N (HN, 11.4 mM N) or low N (LN, 0.55 mM N). Root system architecture was assessed after 9 days. Numbers below each box indicate the number of plants assessed for each genotype under HN and LN. Horizontal lines show medians; box limits indicate the 25th and 75th percentiles; whiskers extend to 1.5 times the interquartile range from the 25th and 75th percentiles. Different letters indicate significant differences at $P < 0.001$ according to one-way ANOVA and post-hoc Tukey test. Scale bars, 1 cm.

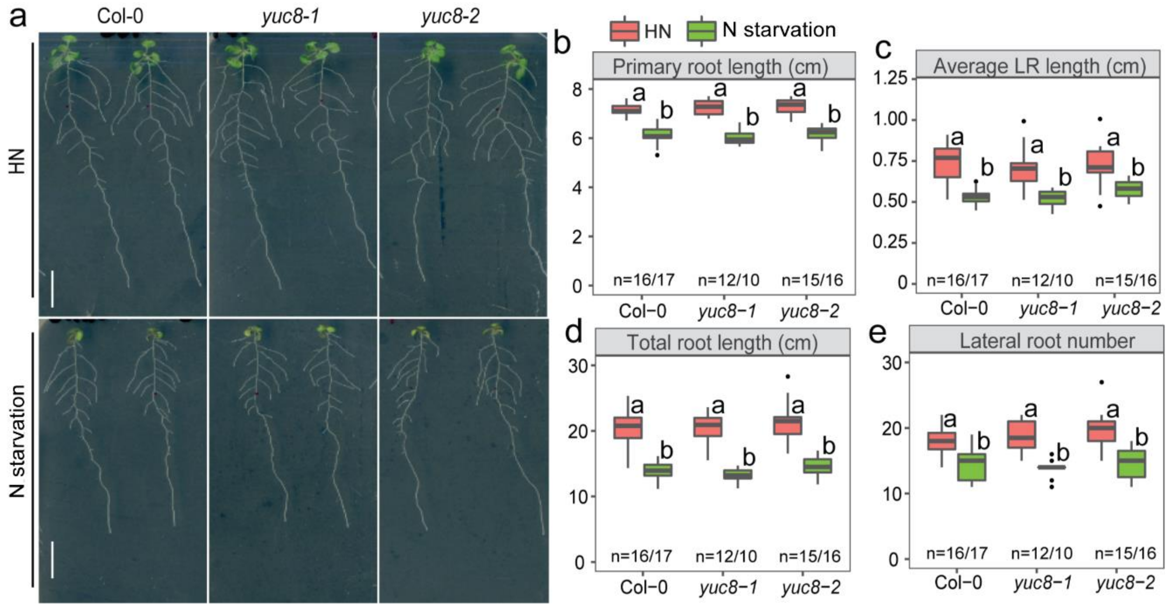

Supplementary Figure 12. Root architectural traits of *yuc8* mutants in response to N starvation. a-e Appearance of plants **(a)**, PR length **(b)**, average LR length **(c)**, total root length **(d)** and LR number **(e)** of wild-type (Col-0) and *yuc8* mutants. Seven-day-old seedlings were pre-cultured on 11.4 mM N and then transferred to agar media containing either high N (HN, 11.4 mM N) or severe low N (severe LN, 0.11 mM N). Root system architecture was assessed after 9 days. Numbers below each box indicate the number of plants assessed for each genotype under HN and severe LN. Horizontal lines show medians; box limits indicate the 25th and 75th percentiles; whiskers extend to 1.5 times the interquartile range from the 25th and 75th percentiles. Different letters indicate significant differences at $P < 0.01$ according to one-way ANOVA and post-hoc Tukey test. Scale bars, 1 cm.

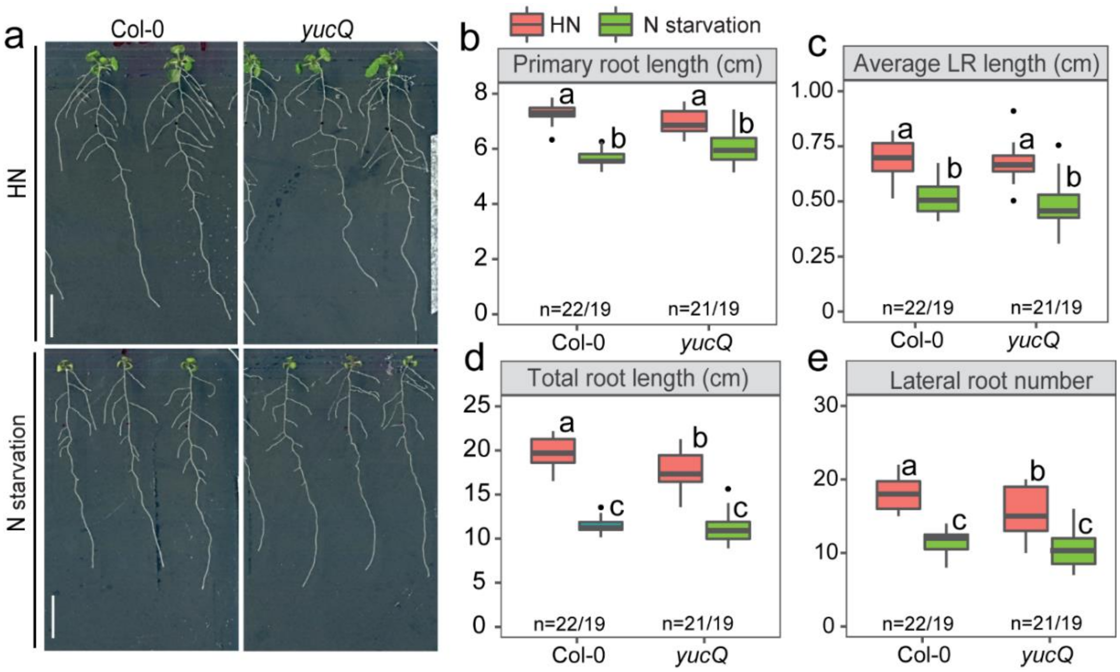

Supplementary Figure 13. Root architectural traits of *yucQ* mutant in response to N starvation. a-e Appearance of plants **(a)**, PR length **(b)**, average LR length **(c)**, total root length **(d)** and LR number **(e)** of wild-type (Col-0) and *yucQ* mutant plants. Seven-day-old seedlings were pre-cultured on 11.4 mM N and then transferred to agar media containing either high N (HN, 11.4 mM N) or severe low N (severe LN, 0.11 mM N). Root system architecture was assessed after 9 days. Numbers below each box indicate the number of plants assessed for each genotype under HN and severe LN. Horizontal lines show medians; box limits indicate the 25th and 75th percentiles; whiskers extend to 1.5 times the interquartile range from the 25th and 75th percentiles. Different letters indicate significant differences at $P < 0.01$ according to one-way ANOVA and post-hoc Tukey test. Scale bars, 1 cm.

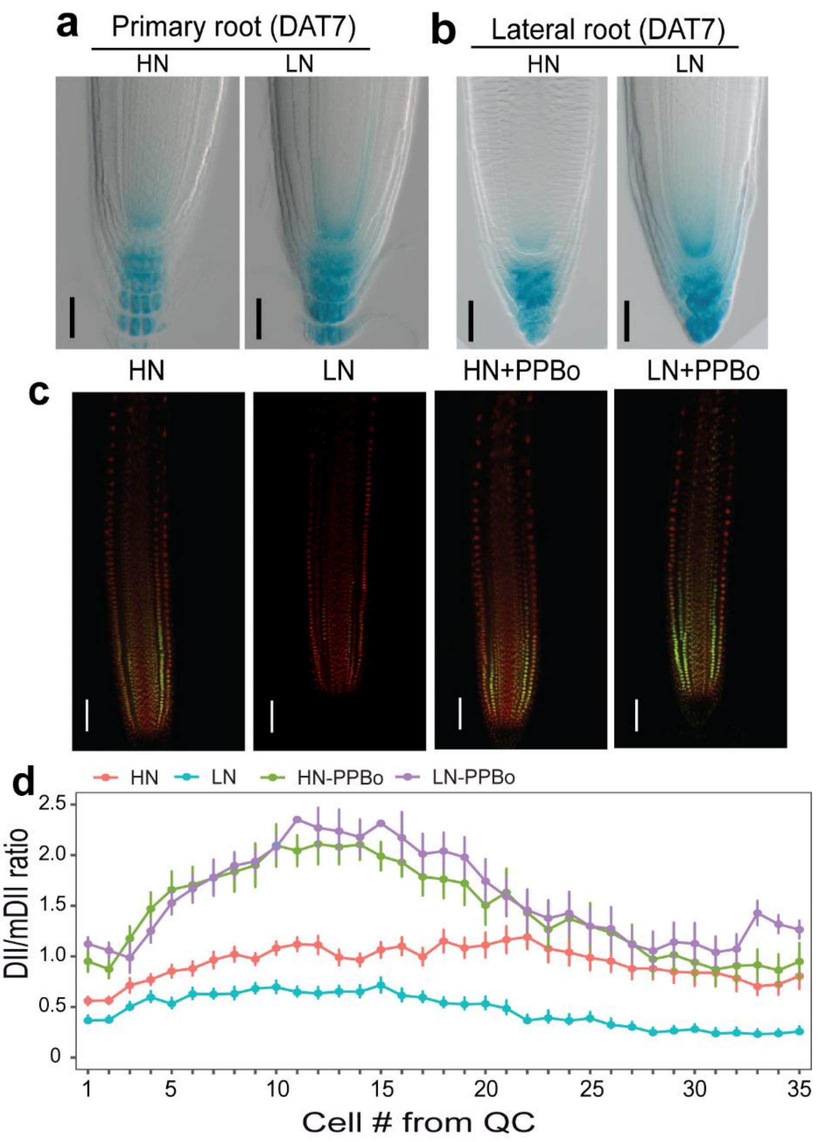

Supplementary Figure 14. Low N increases *YUC8* expression and auxin accumulation in root tips. a-b *proYUC8*-dependent GUS activity in PR **(a)** and LR **(b)** tips at 7 days after transfer (DAT) to high N (HN, 11.4 mM N) or low N (LN, 0.55 mM N). Scale bars, 100 µm. **c** Representative images showing expression of mDII-ntdTomato and DII-n3xVenus in PR tips. Scale bars, 100 µm. **d** DII-n3xVenus/mDII-ntdTomato intensity ratio in epidermal cells of PRs. Seven-day-old seedlings were pre-cultured on 11.4 mM N and then transferred to solid agar containing either HN or LN in the presence or absence of 5 µM YUCCA activity inhibitor p-phenoxyphenylboronic acid (PPBo). PRs were taken for microscopic analyses at 7 days after transfer. The experiment was repeated twice with similar results. Dots represent means ± SEM. Number of individual roots analyzed in HN/LN: *n* = 20/20 (mock) and 15/15 (5 µM PPBo). *P* values for differences between HN vs LN or HN-PPBo vs LN-PPBo according to Welch's *t*-test are presented in Supplementary Fig. 14d in the Source Data File.

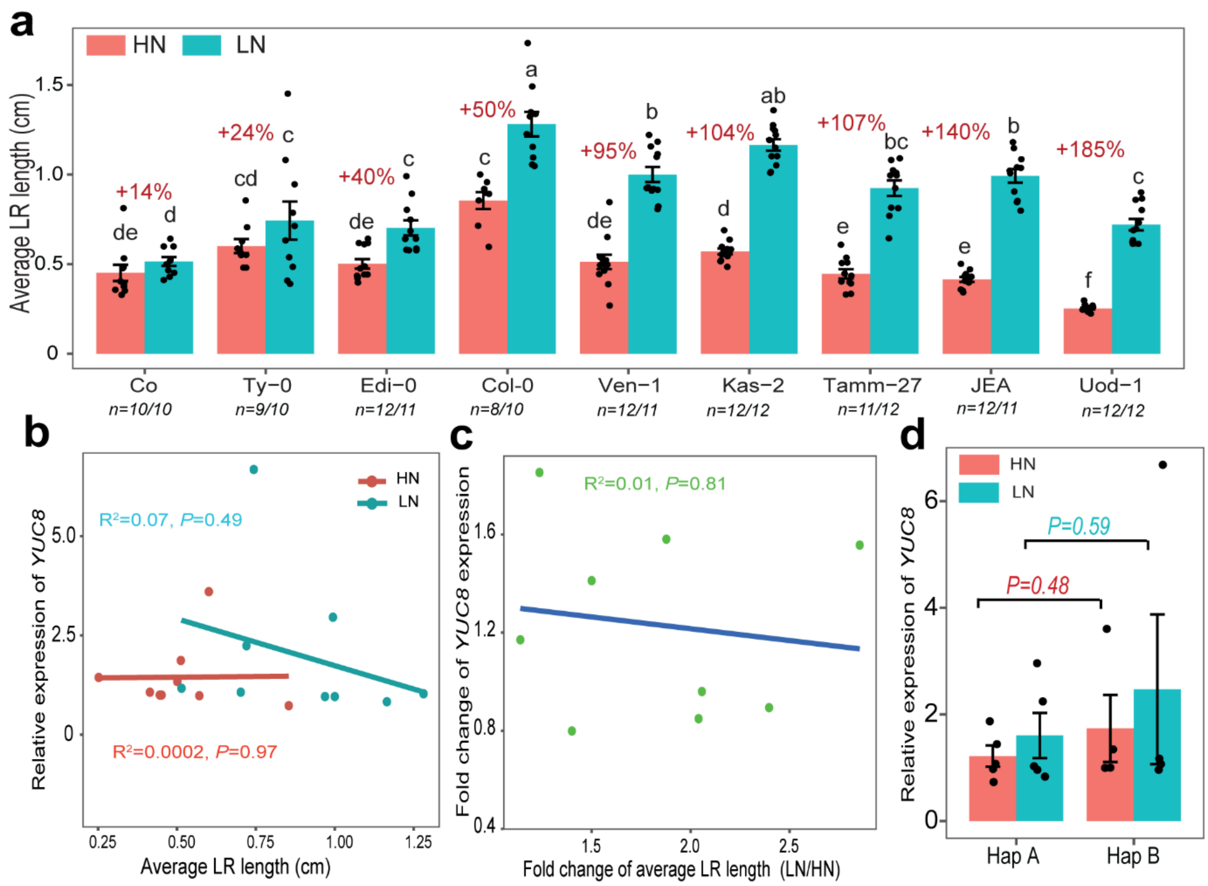

Supplementary Figure 15. *YUC8* expression is not associated with average LR length irrespective of N availability. a Average LR length of nine contrasting natural accessions used for *YUC8* expression analysis**.** Bars represent means ± SEM. Number of individual roots analyzed for each genotype in HN and LN are presented below bars. Different letters indicate significant differences at $P< 0.05$ according to one-way ANOVA and post-hoc Tukey test. Values in red on the top of bars denote the percentage increase of LN/HN LR ratio. **b-c** Correlations between *YUC8* transcript levels **(b)** or their fold change **(c)** in roots and average LR length at two N conditions. **d** Expression level of *YUC8* in accessions from haplotype A (Col-0, Ven-1, Kas-2, JEA, Uod-1) or haplotype B (Co, Ty-0, Edi-0 and Tamm-27). Expression levels were normalized to expression in Co at high N (HN, 11.4 mM N). Bars represent means ± SEM. *P* values relate to differences between haplotype A and haplotype B under HN (in red) or LN (in cyan) according to Welch's *t*-test. No significant differences between haplotype A and haplotype B were detected irrespective of the N condition. Seven-day-old seedlings pre-cultured on 11.4 mM N were transferred to solid agar containing either high N (HN, 11.4 mM N) or low N (LN, 0.55 mM N). Average LR length was determined after 9 days. Root samples for *q*PCR analysis were taken at 9 days after transfer (n=4 independent biological replicates).

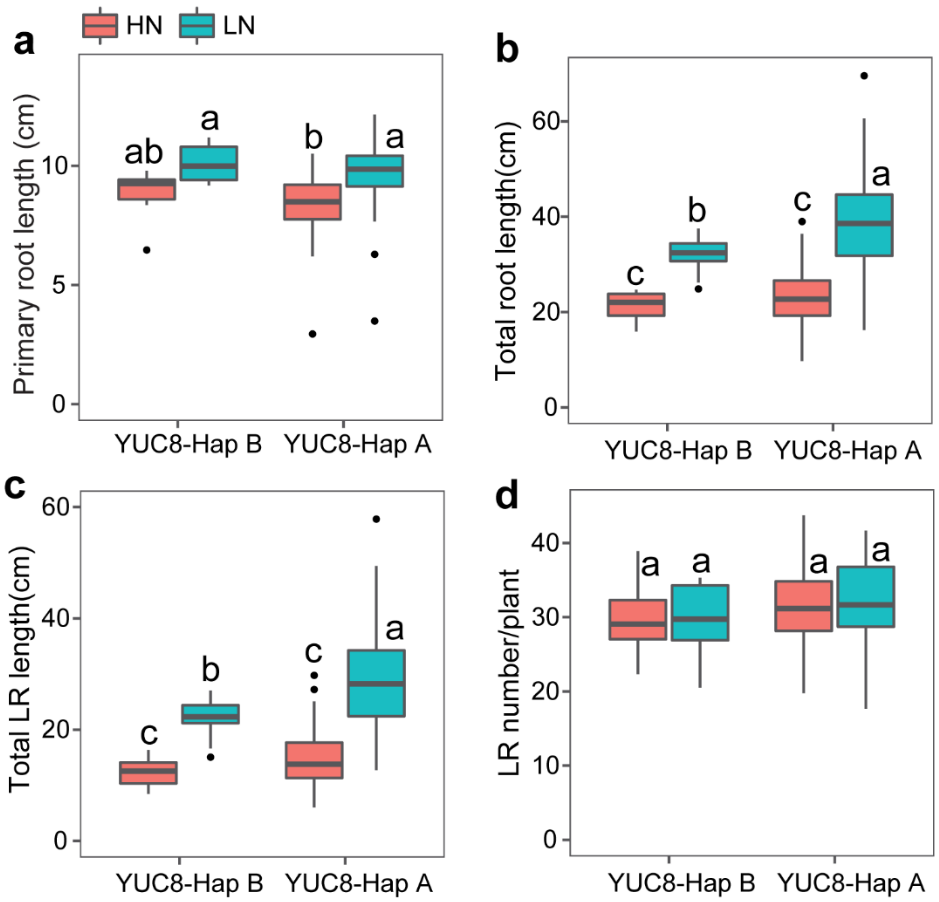

Supplementary Figure 16. YUC8-haplotype A confers longer total LR and total root length under low N than YUC8-haplotype B. a-d, Primary root length (**a**), total root length (**b**), total LR length (**c**) and LR number (**d**) of natural accessions representing two major *YUC8* haplotypes (n = 126 and 10 accessions for haplotype A and haplotype B, respectively). Horizontal lines show medians; box limits indicate the 25th and 75th percentiles; whiskers extend to 1.5 times the interquartile range from the 25th and 75th percentiles. Different letters indicate significant differences at $P < 0.05$ according to one-way ANOVA and post-hoc Tukey test.

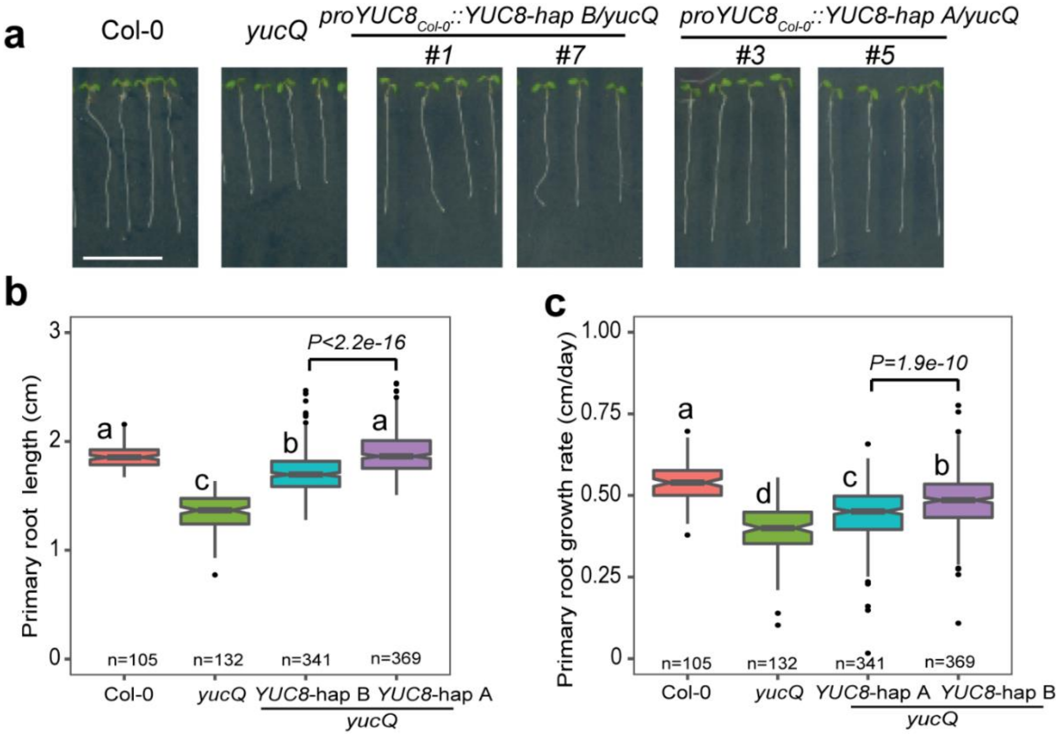

Supplementary Figure 17. Allelic variants of *YUC8* determine distinct root elongation in *yucQ* mutant plants. a Representative photographs of 7-day-old plants of wild-type (Col-0), *yucQ* and independent transgenic plants expressing sequences coding for either the *YUC8*-hap A or *YUC8*-hap B under control of the $YUC8_{Col\text{-}0}$ promoter (two independent representative lines were shown for each construct). Scale bar, 1 cm. **b-c** PR length **(b)** and growth rate **(c)** of 20 independent transgenic T2 lines for each construct. Seeds were germinated on solid media containing 11.4 mM N and 1.5% sucrose. PR length was assessed 7 days after germination and growth rate was calculated by determining PR length of the same plants at 7 and 9 days after germination. Horizontal lines show medians; box limits indicate the 25th and 75th percentiles; whiskers extend to 1.5 times the interquartile range from the 25th and 75th percentiles. Different letters indicate significant differences at $P < 0.00001$ according to one-way ANOVA and post-hoc Tukey test. *P* values relate to differences between YUC8-hapA and YUC8-hapB complementing groups according to Welch's *t*-test.

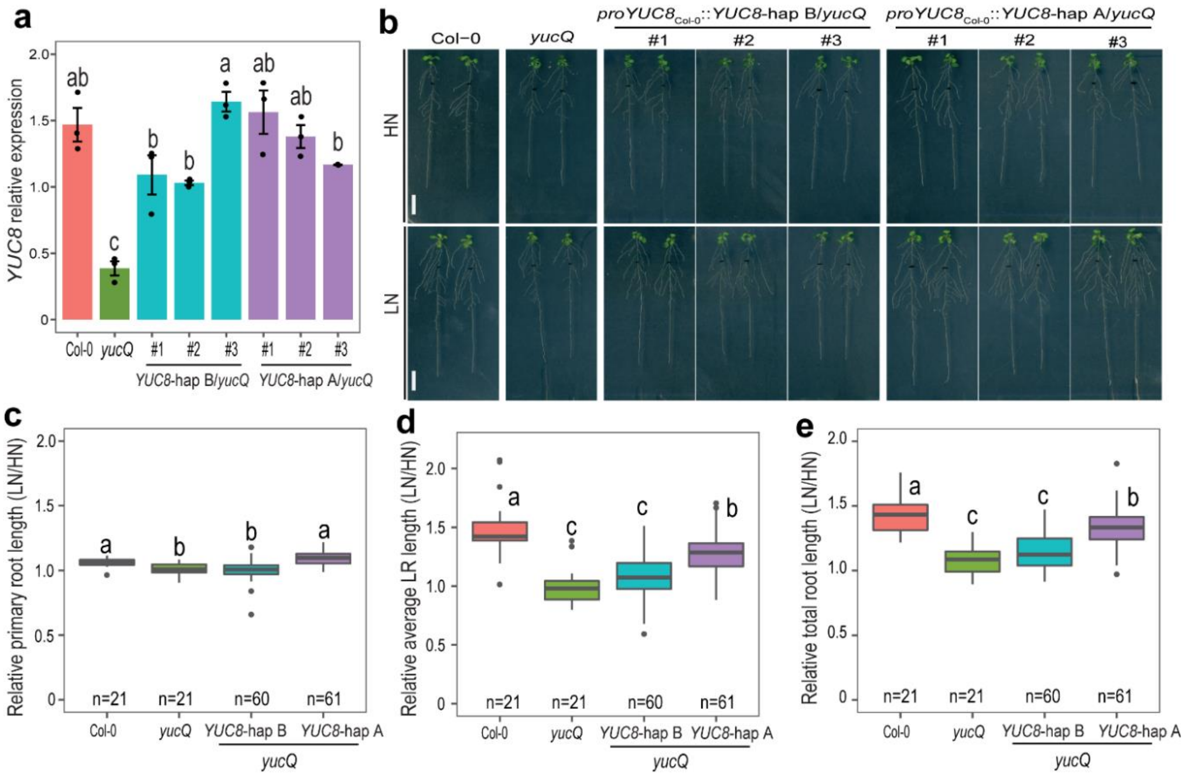

Supplementary Figure 18. *YUC8*-haplotype A confers stronger root foraging responses to low N than *YUC8*-haplotype B. a Transcript levels of *YUC8* in the wild-type (Col-0), *yucQ* and independent T3 homozygous *yucQ* lines complemented with coding sequences of *YUC8*-hap A or -hap B under control of the *YUC8*$_{Col\text{-}0}$ promoter (three independent lines were selected for each construct). Bars represent means ± SEM (n = 3 biological replicates). **b-e** Appearance of plants **(b)**, relative change of PR length **(c)**, average LR length **(d)** and total root length **(e)** in response to low N. Horizontal lines show medians; box limits indicate the 25th and 75th percentiles; whiskers extend to 1.5 times the interquartile range from the 25th and 75th percentiles. Different letters indicate significant differences at P< 0.05 according to one-way ANOVA and post-hoc Tukey test.

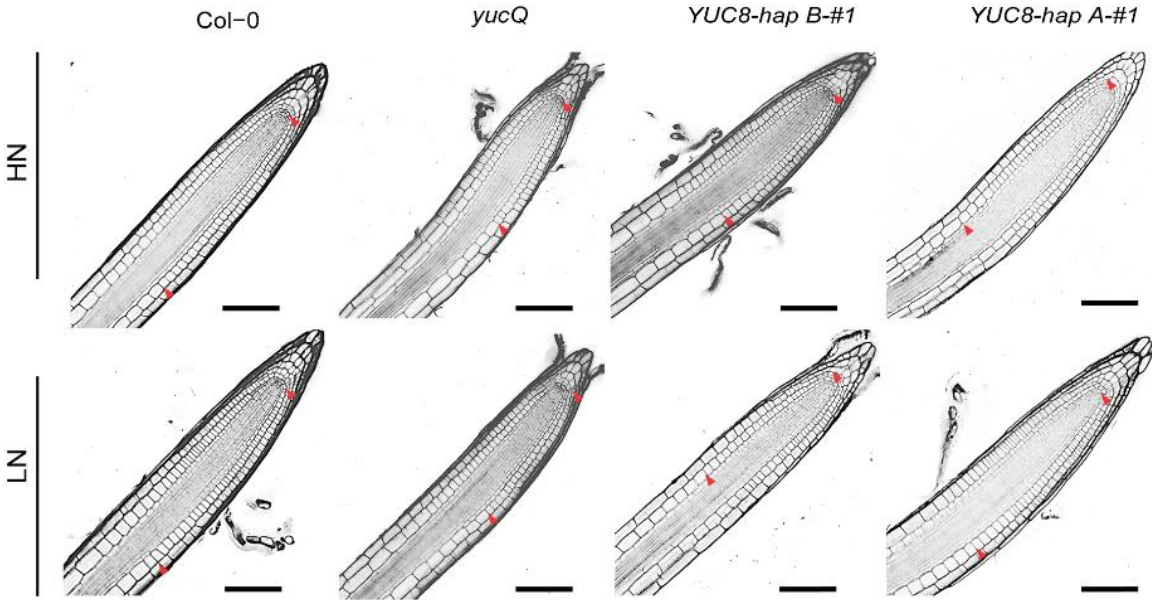

Supplementary Figure 19. Complementation of *yucQ* LR meristem length with two YUC8 variants. Representative confocal images of meristems of mature LRs of wild-type (Col-0), *yucQ* and transgenic lines complemented with either *YUC8* variants under control of the $YUC8_{Col\text{-}0}$ promoter (one representative line shown for each construct). Red arrowheads indicate the position of the quiescent center (QC) and the boundary between the meristematic zone and elongation zone. Scale bars, 100μm. Seven-day-old seedlings were pre-cultured on 11.4 mM N and then transferred to high N (HN, 11.4 mM N) or low N (LN, 0.55 mM N). LR meristems were measured after 9 days and quantitative data are presented in Fig. 4i.

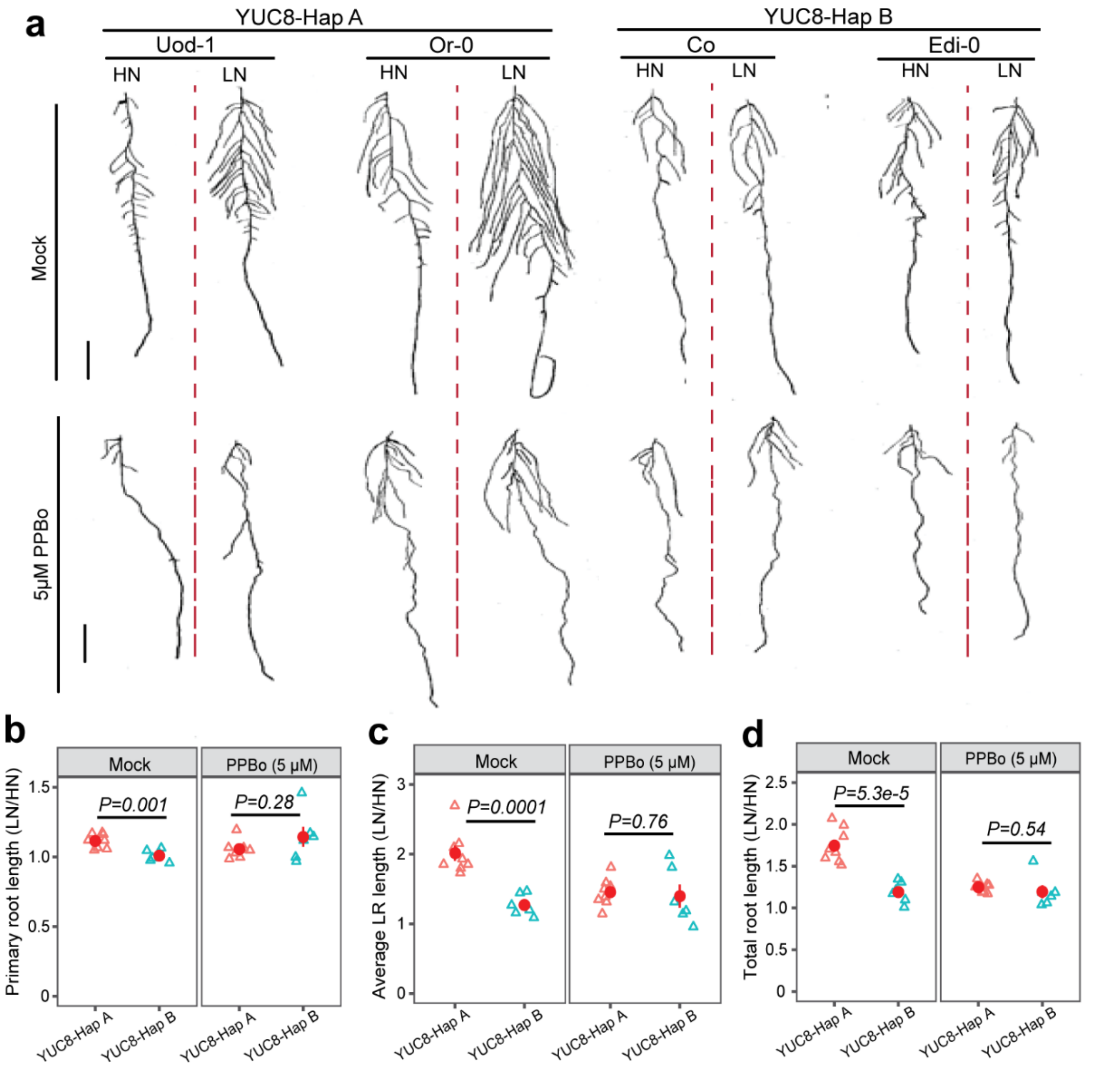

Supplementary Figure 20. Suppression of auxin biosynthesis abolishes the distinct root foraging responses of YUC8-Hap A and YUC8-Hap B accessions. a Phenotypes of two representative accessions of *A. thaliana* belonging to YUC8-Hap A or YUC8-Hap B in response to low N availability and exogenous supply of the YUCCA inhibitor 4-phenoxyphenyl boronic acid (PPBo). **b-d**, LN/HN ratio of PR length **(b)**, average LR length **(c)**, total root length **(d)** of 8 and 6 natural accessions belonging to YUC8-Hap A (Uod-1, Or-0, Wt-5, Kas-2, JEA, Ven-1, Pog-0, Ri-0) and YUC8-Hap B (Co, Edi-0, Tha-1, Ty-0, Alst-1, Tamm-27), respectively. Seven-day-old seedlings were pre-cultured on 11.4 mM N and then transferred to agar media containing either high N (HN, 11.4 mM N) or low N (0.55 mM N) in the presence or absence of 5 µM PPBo. Root system architecture was assessed for at least 7 plants of each genotype and treatment after 9 days. Each triangle represents the mean value of LN-to-HN ratio for each genotype. Dots and crossbars represent means ± SEM (n=8 and 6 for YUC8-Hap A and YUC8-Hap B, respectively). *P* values relate to differences between two haplogroups in Mock or PPBo treatments according to Welch's *t*-test. Scale bars, 1 cm.

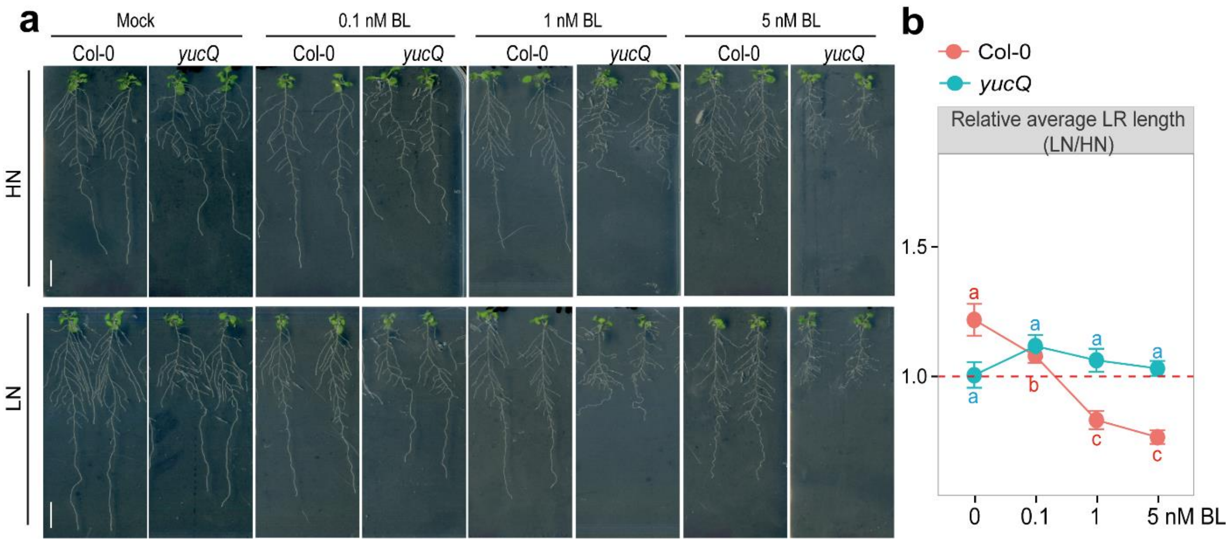

Supplementary Figure 21. Root growth of Col-0 and *yucQ* in response to BR supply under different N availabilities. a-b Appearance of plants **(a)** and relative average LR length **(b)** of Col-0 and *yucQ* plants grown at high N (HN, 11.4 mM N) or low N (LN, 0.55 mM N) in the presence or absence of 24-epibrassinolide (BL)**.** Seven-day-old wild-type (Col-0) and *yucQ* seedlings were pre-cultured on 11.4 mM N and then transferred to solid agar media containing either HN or LN in the presence or absence of the indicated BL concentrations. LR length was assessed after 9 days. Dots represent means ± SEM. Number of individual roots analyzed in Col-0/*yucQ*: n = 9/10 (mock), 10/11 (0.1 nM BL), 9/12 (1 nM BL) and 10/12 (5 nM BL). Different letters indicate significant differences within respective genotypes at $P < 0.05$ according to one-way ANOVA and post-hoc Tukey test. Scale bars, 1 cm.

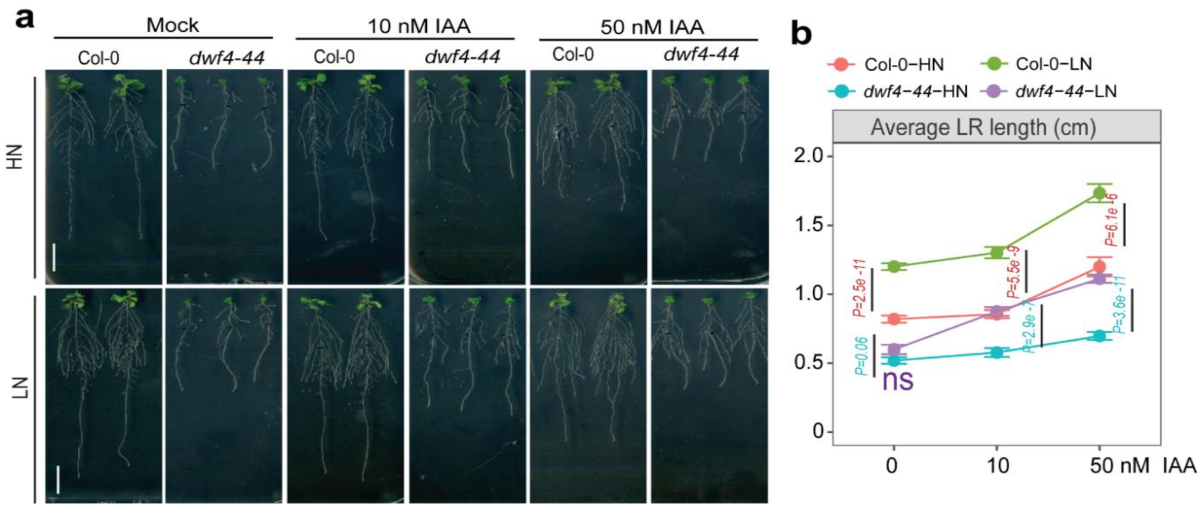

Supplementary Figure 22. Exogenous supply of auxin increases LR length and its response to low N in *dwf4-44*. a-b Appearance of plants **(a)** and average LR length **(b)** of Col-0 and *dwf4-44* plants grown at high N (HN, 11.4 mM N) or low N (LN, 0.55 mM N) in the presence or absence of auxin (in the form of IAA)**.** Seven-day-old wild-type (Col-0) and *dwf4-44* seedlings were pre-cultured on 11.4 mM N and then transferred to solid agar media containing either HN or LN in the presence or absence of indicated concentrations of IAA. LR length was assessed after 9 days. Dots represent means ± SEM. Number of individual roots analyzed in HN/LN: *n* = 14/15 (mock), 12/15 (10 nM IAA), 15/15 (50 nM IAA) for Col-0; *n* = 16/14 (mock), 15/15 (10 nM IAA), 16/17 (50 nM IAA) for *dwf4-44*. *P* values relate to differences between two N conditions for Col-0 (in red) and *dwf4-44* (in cyan) at each IAA treatment according to Welch's *t*-test. Scale bars, 1 cm.

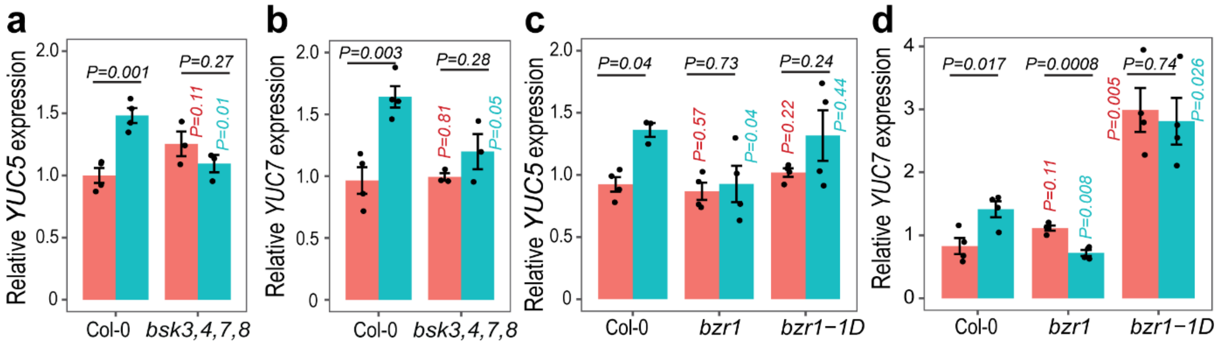

Supplementary Figure 23. *YUC5* and *YUC7* are up-regulated by low N in a brassinosteroid signaling-dependent manner. a-b Transcript levels of *YUC5* **(a)** and*YUC7* **(b)** in wild-type (Col-0) and *bsk3,4,7,8* roots in response to high N (HN, 11.4 mM N) or low N (LN, 0.55 mM N). (**c**-**d**) Transcript levels of *YUC5* **(c)** and*YUC7* **(d)** in wild-type (Col-0), *bzr1* and *bzr1-1D* roots at HN or LN availabilities. 7-day-old seedlings were pre-cultured on 11.4 mM N and then transferred to agar containing either HN or LN. Samples for *q*PCR analysis were taken 9 days after transfer. Expression levels were normalized to *ACT2* and *UBQ10*. Bars represent means ± SEM. (*n* = 4 for Col-0, *bzr1*, *bzr1-1D*, and *3* independent biological replicates for *bsk3,4,7,8* at both N conditions). *P* values relate to differences between two N conditions for each genotype (in black) or between wild-type and mutant plants under HN (in red) or LN (in cyan) according to Welch's *t*-test.

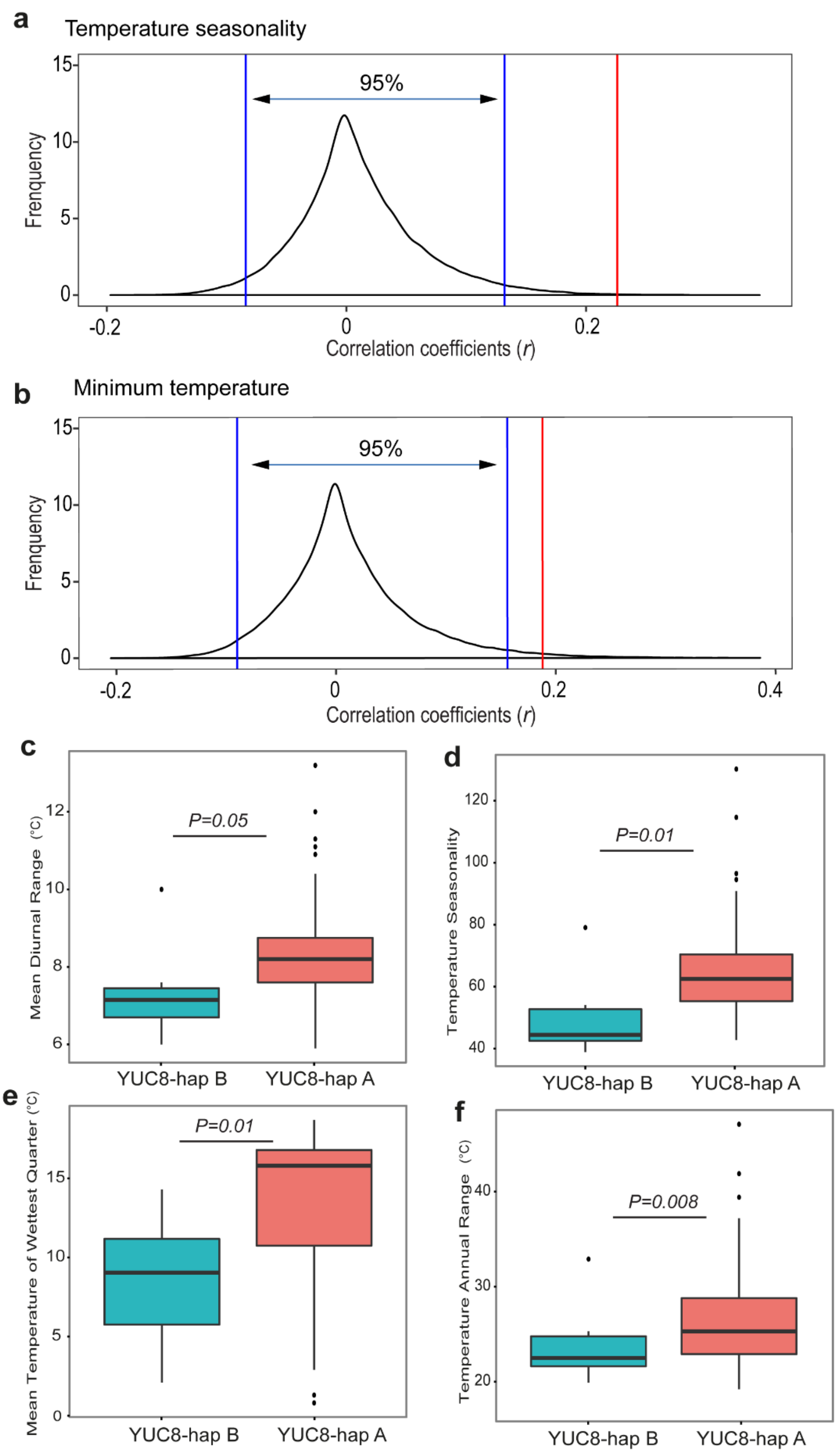

Supplementary Figure 24. Temperature as a possible environmental factor shaping allelic variation in *YUC8*. a-b Distribution of SNP-climate parameter associations for temperature seasonality **(a)** and minimum temperature **(b)** redrawn from data of a GWAS for climate adaptation reported previously (Hancock et al, 2011). x-axis stands for significance of associations (Spearman's correlation coefficients, *r*). y-axis represents frequency of

association coefficients. Area between two blue lines indicate the range encompassing 95% of SNPs. Red lines indicate correlations of the significant SNP identified in GWAS for LR response to low N (SNP_Chr4_14192732), residing outside of the 95% range (r = 0.226 and 0.188 in **a** and **b**, respectively). **c-f** Boxplot showing the association between YUC8-hap A or -hap B and temperature in the geographic location of the accession lines in terms of mean diurnal range **(c)**, temperature seasonality **(d)**, mean temperature of wettest quarter **(e)** or temperature annual range **(f)**. *n* = 105 and 8 accessions for YUC8-hap A and YUC8-hap B, respectively. Horizontal lines show medians; box limits indicate the 25th and 75th percentiles; whiskers extend to 1.5 times the interquartile range from the 25th and 75th percentiles. *P* values between two haplogroups were calculated according to Welch's *t*-test.

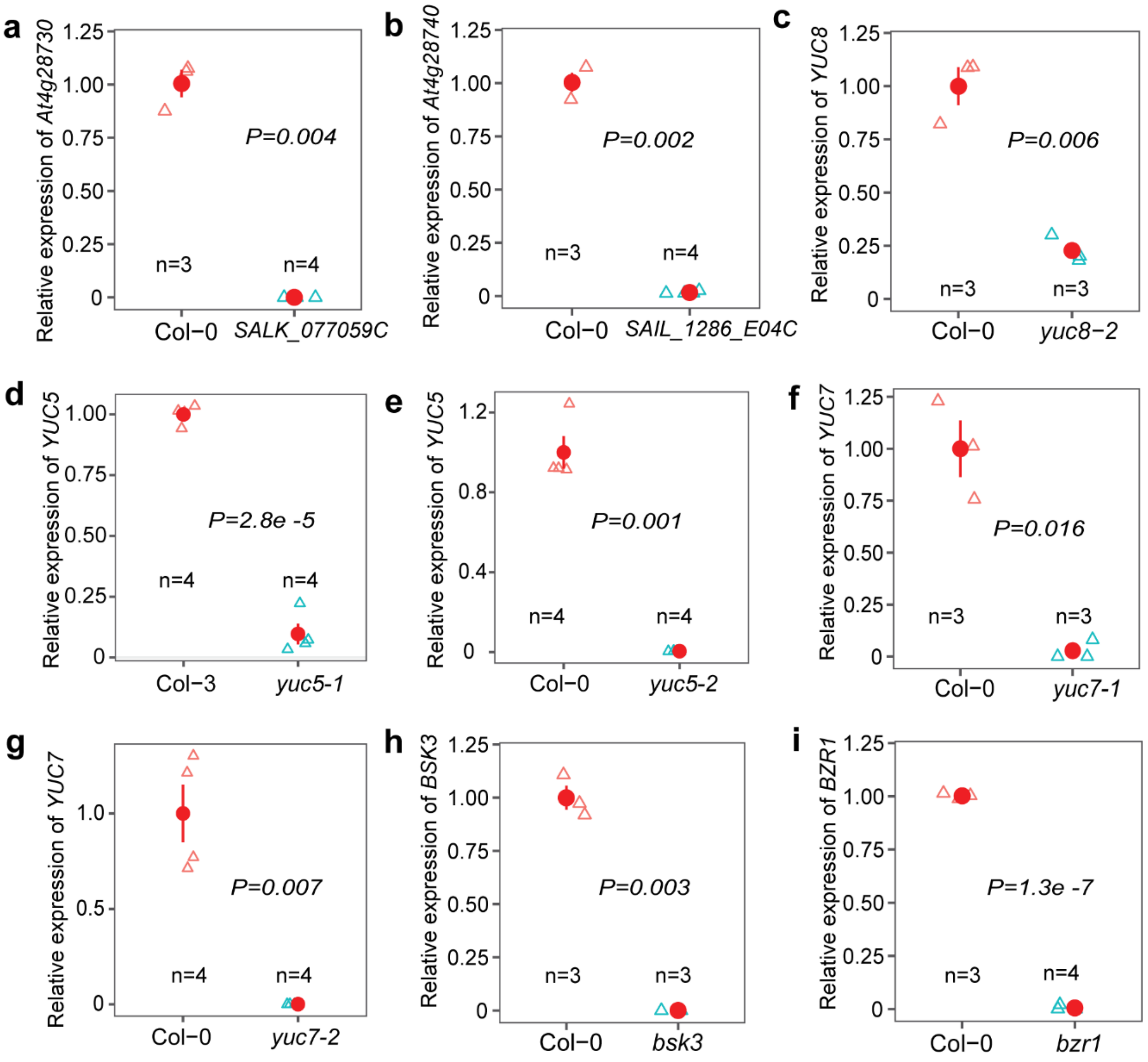

Supplementary Figure 25. Characterization of T-DNA insertion lines used in the study by *q*RT-PCR. a-i Transcript levels of *At4g28730* **(a)**, *At4g28740* **(b)**, *YUC8* **(c)**, *YUC5* **(d,e)**, *YUC7* **(f,g)**, *BSK3* **(h)** and *BZR1* **(i)** in wildtype and corresponding T-DNA insertion lines. Seven-day-old seedlings grown on 11.4 mM N were sampled for *q*PCR analysis. Expression levels were normalized to *ACT2*. Each triangle represents the value of one individual biological replicate. Red dots with crossbars represent means ± SEM. *P* values for differences between wildtype and mutants were calculated according to Welch's *t*-test.
