# Supplementary material for: Local auxin biosynthesis acts downstream of brassinosteroids to trigger root foraging for nitrogen
Source: Nat Commun. 2021 Sep 14;12:5437. doi: 10.1038/s41467-021-25250-x (PMC8440578; doi:10.1038/s41467-021-25250-x)
Supplement: Supplementary file 3 — Description of Additional Supplementary Files [file 41467_2021_25250_MOESM3_ESM.docx]

**Descriptions of Additional Supplementary Files**

**File Name: Supplementary Data 1**

Description: List of 200 geographically referenced Arabidopsis accessions screened for average lateral root length in HN vs LN conditions.

**File Name: Supplementary Data 2**

Description: Single nucleotide polymorphism (SNP, MAF>5%) identified in the coding sequences (CDS) of YUC8 from 139 natural accessions.

**File Name: Supplementary Data 3**

Description: Haplotype analysis of natural YUC8 variants.

**File Name: Supplementary Data 4**

Description: Primers used in the present study.

**File Name: Supplementary Data 5**

Description: Arabidopsis T-DNA insertion lines used in the present study.

**File Name: Supplementary Data 6**

Description: List of 19 climate variables, latitude and longitude of 113 natural accessions divided in two YUC8 haplotypes.
